# SSCMDA: spy and super cluster strategy for MiRNA-disease association prediction

**DOI:** 10.18632/oncotarget.22812

**Published:** 2017-12-01

**Authors:** Qi Zhao, Di Xie, Hongsheng Liu, Fan Wang, Gui-Ying Yan, Xing Chen

**Affiliations:** ^1^ School of Mathematics, Liaoning University, Shenyang, China; ^2^ Research Center for Computer Simulating and Information Processing of Bio-Macromolecules of Liaoning Province, Shenyang, China; ^3^ School of Life Science, Liaoning University, Shenyang, China; ^4^ School of Mechatronic Engineering, China University of Mining and Technology, Xuzhou, China; ^5^ Jiangsu Key Laboratory of Mine Mechanical and Electrical Equipment, China University of Mining and Technology, Xuzhou, China; ^6^ Academy of Mathematics and Systems Science, Chinese Academy of Sciences, Beijing, China; ^7^ School of Information and Control Engineering, China University of Mining and Technology, Xuzhou, China

**Keywords:** microRNA, disease, association prediction, spy strategy, super cluster strategy

## Abstract

In the biological field, the identification of the associations between microRNAs (miRNAs) and diseases has been paid increasing attention as an extremely meaningful study for the clinical medicine. However, it is expensive and time-consuming to confirm miRNA-disease associations by experimental methods. Therefore, in recent years, several effective computational models for predicting the potential miRNA-disease associations have been developed. In this paper, we proposed the Spy and Super Cluster strategy for MiRNA-Disease Association prediction (SSCMDA) based on known miRNA-disease associations, integrated disease similarity and integrated miRNA similarity. For problems of mixed unknown miRNA-disease pairs containing both potential associations and real negative associations, which will lead to inaccurate prediction, spy strategy is adopted by SSCMDA to identify reliable negative samples from the unknown miRNA-disease pairs. Moreover, the super-cluster strategy could gather as many positive samples as possible to improve the accuracy of the prediction by overcoming the shortage of lacking sufficient positive training samples. As a result, the AUCs of global leave-one-out cross validation (LOOCV), local LOOCV and 5-fold cross validation were 0.9007, 0.8747 and 0.8806+/-0.0025, respectively. According to the AUC results, SSCMDA has shown a significant improvement compared with some previous models. We further carried out case studies based on various version of HMDD database to test the prediction performance robustness of SSCMDA. We also implemented case study to examine whether SSCMDA was effective for new diseases without any known associated miRNAs. As a result, a large proportion of the predicted miRNAs have been verified by experimental reports.

## INTRODUCTION

MicroRNAs (miRNAs) are non-coding RNAs which are composed of approximately 22 nucleotides [1]. The miRNAs can influence some important biological process, such as cell development, proliferation, and apoptosis [2]. As an important research result, the miRNAs have been shown to be responsible for the regulation of approximately 60% of the coding genes in mammalian [3]. Furthermore, their regulatory functions have also been shown to be related to some special gene expressions in the post transcription stage [4]. For example, they can inhibit translation or cause miRNA degradation of target genes to repress gene expression [5]. The first miRNA was found about twenty years ago, after that a large amount of miRNAs have been discovered from a wide variety of species including human [6, 7]. Furthermore, increasing numbers of miRNAs have been experimentally shown to be associated with the development processes of various human diseases [8, 9]. For example, the study of Benjamin *et al*. has identified differentially expressed miRNAs between malignant pleural mesothelioma (MPM) and various carcinomas using microarrays. Among them, hsa-miR-193-3p was over-expressed in MPM, while hsa-miR-200c and hsa-miR-192 were over-expressed in peripheral lung adenocarcinoma and carcinomas that frequently metastasize to lung pleura [10]. Additionally, in the study of William *et al*. in 2010, they demonstrated the critical role of miR-155 in regulation of cell survival and chemosensitivity through down-regulation of FOXO3a in breast cancer [11]. Moreover, Wang *et al*. confirmed that the concentrations of plasma miR-17-5p/20a were significantly associated with the differentiation status and TNM stages of gastric cancer, where the TNM is a notation system that describes the stage of a cancer which originates from a solid tumour with alphanumeric codes [12]. Though so many associations between miRNAs and diseases have been discovered, the known associations are only the tip of the iceberg. As far as we know, a large amount of experiments has been implemented for discovering their potential associations because the identification of miRNA-disease association has great significance for the diagnosis and treatment of human complex diseases. However, experimental methods are expensive and time-consuming. Therefore, increasing studies have focused on the computational algorithms to predict the probabilities of the potential miRNA-disease associations [13–26]. By choosing the most promising associated miRNAs, experiment methods could be more effective [27, 28].

Based on the assumption that functionally similar miRNAs are more likely to have relevance to diseases with similar phenotypic traits, Jiang *et al*. [29] proposed a hypergeometric distribution-based model which could provide the prediction of miRNA-disease associations through the disease phenotype similarity network, miRNA functional similarity network, and known human disease-miRNA association network. However, this computational model strongly relies on predicted miRNA-target interactions which have high rates of false-positive and false-negative prediction results. Shi *et al*. [30] introduced a modified random walk algorithm by taking advantage of the miRNA-target interactions, disease-gene associations and protein-protein interactions (PPIs) to acquire potential associations between the miRNAs and diseases. Mork *et al*. [31] presented a miRNA-Protein-Disease based miRNA-disease association prediction (miRPD) method through combining the protein-disease associations with the protein-miRNA interactions based on the shared proteins between miRNAs and the diseases to provide miRNA-disease association prediction scores. Xu *et al*. [32] constructed a miRNA prioritization method by integrating known disease-gene associations and miRNA-target interactions. Instead of using the known miRNA-disease associations, this method analyzed the similarity between the targets of miRNAs and disease genes. All these aforementioned methods could not generate sufficient accurate prediction results, because they strongly rely on the predicted miRNA-target interactions, which have high rates of false positive and false negative. Another reason is that current disease-gene association network is incomplete.

Several computational methods were proposed to overcome limitations of the previous methods. Xuan *et al*. [33] constructed an effective miRNA-disease association prediction model named HDMP based on weighted k most similar neighbors by assigning higher weights to similarity scores between members in the same miRNA cluster or family when calculating miRNA similarity. However, the HDMP was not suitable for detecting the potential association with respect to new diseases or new miRNAs. Additionally, HDMP could not perform better than most of the previous models that were calculated based on the global network similarity measures. Chen *et al*. [34] were the first to propose the global-network-similarity-based computational model called Random Walk with Restart for MiRNA-Disease Association prediction (RWRMDA) based on the global information of human miRNA functional similarity network and known human miRNA-disease association network. RWRMDA performed well in the cross validations and case studies on several human complex diseases. However, its unavailability for new diseases without any known related miRNAs was still a certain limitation. Chen *et al*. [20] presented another model of Within and Between Score for MiRNA-Disease Association prediction (WBSMDA) to predict potential miRNA-disease associations based on miRNA-disease associations, integrated miRNA similarity and integrated disease similarity. WBSMDA could predict the potential related miRNAs for new diseases without any known related miRNAs and new miRNAs without any known associated diseases, which could overcome the limitation of RWRMDA and HDMP. Chen *et al*. [35] also developed a Heterogeneous Graph Inference model for MiRNA-Disease Association prediction (HGIMDA) by using an iterative process based on global network similarity information to find the optimal solutions, which had better performances in comparison with the previous models. Li *et al.* [36] developed a matrix completion for miRNA-disease association prediction model (MCMDA) based on the known miRNA-disease associations in the Human MicroRNA Disease Database (HMDD). MCMDA used the singular value thresholding (SVT) algorithm to accomplish the matrix completion procedure. It obtained excellent performances although it merely depended on known miRNA-disease associations.

More and more studies have taken focus on the using of machine learning methods to predict associations between miRNAs and diseases [37–40]. Among those studies, Xu *et al*. [41] proposed a miRNA-target dysregulated network (MTDN) through combining miRNA-target interactions and expression pattern of miRNAs and mRNAs. They constructed the support vector machine (SVM) classifier to distinguish positive miRNA-disease associations from negative samples based on the network topologic information feature. However, it was hard to obtain the true negative miRNA-disease associations, which influenced the accuracy of the supervised classifier. Chen *et al*. [42] used semi-supervised learning to construct the Regularized Least Squares for MiRNA-Disease Association prediction (RLSMDA) model with disease semantic similarity, miRNA functional similarity and known miRNA-disease associations. RLSMDA could calculate the associated probabilities of related miRNAs for diseases without any known associated miRNAs. Meanwhile, it could avoid the problem of using negative associations between miRNAs and diseases. However, the choice of parameters for RLSMDA and the ways of combining the classifiers in different spaces together could influence the accuracy of prediction results. Furthermore, Chen *et al*. [14] developed the Restricted Boltzmann Machine for Multiple types of MiRNA-Disease Association prediction (RBMMMDA) method based on miRNA-disease associations. This model presented restricted Boltzmann machine (RBM) with a two-layer (visible and hidden) undirected graph RBMMMDA could obtain both new miRNA-disease associations and their corresponding association types.

In this paper, we proposed Spy and Super Cluster strategy for MiRNA-Disease Association prediction (SSCMDA) model based on the Regularized Least Square (RLS) classifier to predict the potential miRNA-disease associations. To fully integrate the known information, we used the known miRNA-disease associations, integrated miRNA similarity and integrated disease similarity as our input data. Firstly, we took spy samples from positive samples to determine potential negative samples with a high degree of confidence. Furthermore, the super cluster strategy has been used to increase as many positive samples as possible to improve the prediction accuracy. According to the results of cross validation, the areas under the ROC curves (AUCs) of global and local leave-one-out cross validation (LOOCV) were 0.9007 and 0.8747, respectively. The 5-fold cross validation showed an average AUC of 0.8806+/-0.0025. For further validation, we carried out the case studies based on known miRNA-disease associations in the latest version and previous version of HMDD database to test whether the predicted associations had been verified by reports in other databases, respectively. We also implemented case studies for new diseases that did not have any known related miRNAs. As a result, there were high proportions of the predicted miRNAs confirmed by recent experimental reports. According to the performances of all the evaluation methods implemented above, SSCMDA has shown anticipated prediction accuracy.

## RESULTS

### Performance evaluation

**Figure 1:**
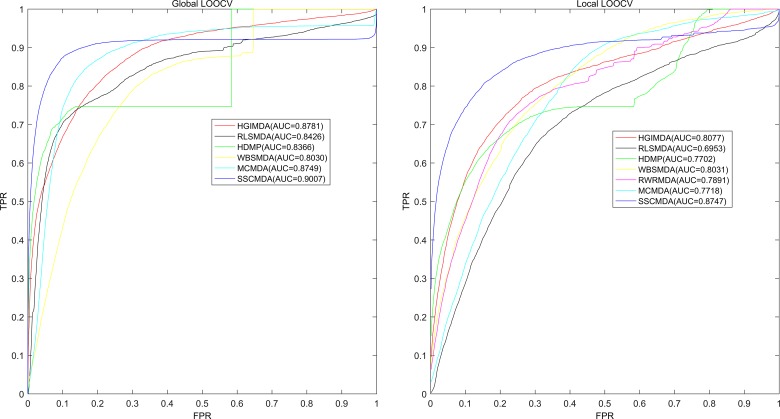
AUC of global LOOCV (left) compared with MCMDA, HGIMDA, RLSMDA, HDMP and WBSMDA; AUC of local LOOCV (right) compared with MCMDA, HGIMDA, RLSMDA, HDMP, WBSMDA and RWRMDA As a result, SSCMDA achieved AUCs of 0. 9007 and 0.8747 in the global and local LOOCV, which exceed all the previous classical models.

As is known, the receiver operating characteristic curve (ROC) is popular to indicate the evaluation performance of the prediction accuracy based on corresponding cross validations. Therefore, we drew ROC curve to present the visible accuracy description [43]. Through plotting the true positive rate (TPR, sensitivity) versus the false positive rate (FPR, 1-specificity) at different thresholds, we obtained the curve that is closer to the upper left corner to mean the better prediction effectiveness. Specifically, sensitivity refers to the percentage of the true positive samples whose rankings are higher than the given threshold in the whole positive samples. Meanwhile, specificity denotes the percentage of negative samples with rankings lower than the threshold in the whole negative samples. Furthermore, AUC was calculated to demonstrate the prediction ability of SSCMDA. If the AUC equals to 1, it indicates that the model has perfect prediction performance. If the AUC equals to 0.5, it indicates that the model only has a random prediction performance. According to the validation index mentioned above, we firstly used global LOOCV based on the known miRNA-disease associations in latest version of HMDD database to evaluate the accuracy of SSCMDA. In each round, we changed one of the known miRNA-disease associations to candidate one which was deemed as unknown miRNA-disease pair. Then we used the SSCMDA to predict all the probability scores of the candidate pairs, and compared the score of the test sample which was changed above with the scores of other candidate pairs to observe whether it ranked above the given threshold. Different from the global LOOCV, local LOOCV just considered miRNAs that were associated with the investigated disease. Specifically, we compared the score of the test sample with the scores of candidate miRNA-disease pairs that were just related to the investigated disease. As a result, SSCMDA obtained the AUC of 0.9007 in the global LOOCV and AUC of 0.8747 in the local LOOCV as shown in Figure 1. In comparison with some previous models, the SSCMDA has really shown an improvement of the prediction accuracy in some extent. For example, the AUCs obtained from MCMDA, HGIMDA, RLSMDA, HDMP, WBSMDA were 0.8749, 0.8781, 0.8426, 0.8366, 0.8030 in global LOOCV and 0.7718, 0.8077, 0.6953, 0.7702, 0.8031 in local LOOCV, respectively. However, RWRMDA only had AUC of local LOOCV (0.7891) which was a defect because it could not uncover the missing associations for all the diseases simultaneously. In conclusion, the proposed model SSCMDA performed well in the global LOOCV and local LOOCV (See Figure 1). To show the comparison with a more clear form, we also concluded the global and local Growth Rate (GR) of SSCMDA compared with the previous models and show them in Table 1 together with their AUCs of global and local LOOCV (See Table 1).

**Table 1:** Comparison of the proposed method with all the previous methods according to the AUCs of global and local LOOCV and their Growth Rate (GR) of SSCMDA compared with the other models

Model	Global LOOCV	Global GR of SSCMDA	Local LOOCV	Local GR of SSCMDA
SSCMDA	0.9007	——	0.8747	——
HGIMDA	0.8781	2.57%	0.8077	8.30%
RLSMDA	0.8426	6.90%	0.6953	25.80%
HDMP	0.8366	7.66%	0.7702	13.57%
WBSMDA	0.8030	12.17%	0.8031	8.92%
RWRMDA	——	——	0.7891	10.85%
MCMDA	0.8749	2.95%	0.7718	13.33%

We also carried out 5-fold cross validation for a further evaluation for the accuracy of the SSCMDA model. Firstly, we randomly divided the whole known associations equally into five sections and treated each section as test samples in turn by removing the associations of these test samples simultaneously. The miRNA-disease pairs without known association evidences were regarded as candidate pairs. Afterwards, all the test samples would be scored and compared with the scores of candidate pairs. We repeated the procedure of 5-fold cross validation 100 times randomly to obtain the average AUC value. In comparison with MCMDA, RLSMDA, HDMP and WBSMDA, whose average AUCs were 0.8767+/-0.0011, 0.8569+/-0.0020, 0.8342+/-0.0010 and 0.8185+/-0.0009, we could further confirm the effectiveness of SSCMDA for potential miRNA-disease association prediction with the average AUC of 5-fold cross validation 0.8806+/-0.0025.

### Case Studies

We studied three different typical cases that were case studies for known diseases, case study for new disease, and case study based on old data. We used a total of five major human diseases including breast neoplasm, esophageal neoplasm, lymphoma, hepatocellular carcinoma and glioblastoma to test the prediction effect of the proposed model in all the types of case studies. To present the results of case studies, we further counted the number of the miRNAs verified by at least one database in the top 10, top 20 and even top 50 prediction results of the investigated disease-related miRNAs. For the first case, the numbers of verified miRNAs related with breast neoplasm, esophageal neoplasm and lymphoma in top 50 were 42, 40 and 39, respectively. For the second case, the number of verified miRNAs related with hepatocellular carcinoma in top 50 was 41. For the third case, the number of verified miRNAs related with Glioblastoma in top 50 was 29.

### Case studies for known diseases

We have studied three known diseases based on the confirmation from the other two miRNA-disease association databases dbDEMC [44] and miR2Disease [45] to examine the effectiveness of predicting miRNAs by SSCMDA.

Breast neoplasm is the most common malignancy for women's health, causing large amounts of death each year. The breast neoplasms consist of multiple types of breast neoplasm cells, but only a minority of breast neoplasm cells had the ability to form new tumors [46]. In the western world, over eighty percent of breast neoplasms are hormone-receptor positive [47]. Nowadays, lots of scientists have paid attention to the original etiology of breast cancers in the perspective of miRNAs. Increasing numbers of evidences showed that some miRNAs were highly correlated with breast neoplasm and played important roles in the development of breast neoplasm. For example, among the differentially expressed miRNAs, miR-10b, miR-125b, miR145, miR-21, and miR-155 emerged to be the most consistently deregulated in breast neoplasm. Three of them, miR-10b, miR-125b, and miR-145, were down-regulated and the remaining two, miR-21 and miR-155, were up-regulated, suggesting that they may potentially act as tumor suppressor genes or oncogenes, respectively [48]. Through implementing SSCMDA, we obtained the total ranking of the unknown miRNA-disease pairs. As the result shown, among the top 10, 20 and 50 potential associations between breast neoplasm and miRNAs, there were 9, 19 and 42 miRNA-disease associations confirmed by experiments, respectively (See Table 2). Taking the hsa-mir-215 as an example, which was ranked the first in our prediction results, recent study had shown that its difference in expression was observed between serum samples from healthy volunteers and serum samples from untreated patients with metastatic breast cancer [49].

**Table 2:** Prediction of the top 50 predicted miRNAs associated with breast neoplasms based on known associations in HMDD database

miRNA	Evidence	miRNA	Evidence
hsa-mir-215	dbdemc	hsa-mir-18a	dbdemc;miR2Disease
hsa-mir-15b	dbdemc	hsa-mir-590	dbdemc
hsa-mir-16	dbdemc	hsa-mir-143	dbdemc;miR2Disease
hsa-mir-29b	dbdemc;miR2Disease	hsa-mir-675	unconfirmed
hsa-mir-221	dbdemc;miR2Disease	hsa-mir-326	dbdemc
hsa-mir-15a	dbdemc	hsa-mir-194	dbdemc
hsa-mir-345	dbdemc	hsa-mir-218	dbdemc
hsa-mir-99b	dbdemc	hsa-mir-183	dbdemc
hsa-mir-483	dbdemc	hsa-mir-1302	unconfirmed
hsa-let-7g	dbdemc	hsa-mir-922	unconfirmed
hsa-mir-150	dbdemc	hsa-mir-202	dbdemc;miR2Disease
hsa-let-7c	dbdemc	hsa-mir-195	dbdemc;miR2Disease
hsa-mir-498	dbdemc	hsa-mir-181	unconfirmed
hsa-let-7b	dbdemc	hsa-mir-765	dbdemc
hsa-let-7f	dbdemc;miR2Disease	hsa-mir-100	dbdemc
hsa-mir-145	dbdemc;miR2Disease	hsa-mir-30d	dbdemc
hsa-mir-92	dbdemc	hsa-mir-1323	unconfirmed
hsa-mir-1247	unconfirmed	hsa-mir-200b	dbdemc;miR2Disease
hsa-let-7d	dbdemc;miR2Disease	hsa-let-7a	dbdemc;miR2Disease
hsa-let-7e	dbdemc	hsa-mir-103a	unconfirmed
hsa-mir-223	dbdemc	hsa-mir-10b	dbdemc;miR2Disease
hsa-mir-200a	dbdemc;miR2Disease	hsa-mir-9	dbdemc;miR2Disease
hsa-mir-29a	dbdemc	hsa-mir-17	miR2Disease
hsa-mir-198	dbdemc	hsa-mir-2355	unconfirmed
hsa-mir-25	dbdemc	hsa-mir-141	dbdemc;miR2Disease

The first column records top 1-25 related miRNAs. The second column records the top 26-50 related miRNAs.

Esophageal neoplasm is a deadly cancer but rarely studied worldwide. Additionally, the esophageal neoplasm is age-specific which means that the incidence and mortality rates increased with age [50]. Through the efforts of researchers, great development has been achieved in the epidemiologic patterns associated with this disease during the past three decades. Recent advances in the diagnosis, staging, treatment and prognosis of esophageal neoplasm have led to small but significant improvements in survival [51]. Furthermore, recent researches have shown that the expression of miRNAs has tight associations with the development of esophageal neoplasm. For example, low expression of let-7b and let-7c in biopsies from 74 untreated patients of the training set significantly correlated with poor response to chemotherapy of both clinic and histopathology [52]. Based on the aforementioned facts, SSCMDA was implemented to identify potential related miRNAs for esophageal neoplasm based on known associations in the HMDD database. As a result, 9 out of the top 10 and 40 out of the top 50 predicted associations between esophageal neoplasm and miRNAs were confirmed by experimental reports from dbDEMC database and miR2Disease database (See Table 3). For example, hsa-mir-150 was ranked third among the predicted potential associations and it has been verified to regulate the EMT-inducer ZEB1 in esophageal squamous cell carcinoma [53].

**Table 3:** Prediction of the top 50 predicted miRNAs associated with esophageal neoplasm based on known associations in HMDD database

miRNA	Evidence	miRNA	Evidence
hsa-mir-215	dbdemc	hsa-mir-106b	dbdemc
hsa-mir-15b	dbdemc	hsa-mir-191	dbdemc
hsa-mir-150	dbdemc	hsa-mir-222	dbdemc
hsa-mir-675	unconfirmed	hsa-mir-1915	unconfirmed
hsa-mir-15a	dbdemc	hsa-mir-18b	dbdemc
hsa-mir-141	dbdemc	hsa-mir-22	dbdemc
hsa-mir-146a	dbdemc	hsa-mir-10b	dbdemc
hsa-let-7a	dbdemc	hsa-mir-29b	dbdemc
hsa-mir-143	dbdemc	hsa-mir-155	dbdemc
hsa-mir-200b	dbdemc	hsa-mir-26b	dbdemc
hsa-mir-29a	dbdemc	hsa-mir-590	dbdemc
hsa-mir-17	dbdemc	hsa-mir-301b	unconfirmed
hsa-mir-198	dbdemc	hsa-mir-26a	dbdemc
hsa-mir-125a	dbdemc	hsa-mir-92a	unconfirmed
hsa-mir-2355	unconfirmed	hsa-mir-28	dbdemc
hsa-mir-1247	unconfirmed	hsa-mir-34b	dbdemc
hsa-mir-100	dbdemc	hsa-mir-130b	dbdemc
hsa-mir-219	unconfirmed	hsa-mir-520b	dbdemc
hsa-mir-145	dbdemc	hsa-mir-192	dbdemc;miR2Disease
hsa-mir-450b	unconfirmed	hsa-let-7c	dbdemc
hsa-mir-20a	dbdemc	hsa-mir-335	dbdemc
hsa-mir-765	dbdemc	hsa-mir-181b	dbdemc
hsa-mir-345	dbdemc	hsa-mir-181	unconfirmed
hsa-mir-328	dbdemc	hsa-mir-527	dbdemc
hsa-mir-1302	unconfirmed	hsa-mir-373	dbdemc;miR2Disease

The first column records top 1-25 related miRNAs. The second column records the top 26-50 related miRNAs.

Lymphoma is a form of cancer which encompasses a variety of cancers specific to the lymphatic system [54]. Some of the cells in the lymphatic system grow abnormally and are out of control when lymphoma occurs [54]. Eventually, they may form a tumor whose cells will continue to grow as the cancerous cells and will also continue to reproduce [54]. If all these cancerous cells are the same, they are called malignant or cancerous, because they will continue to grow and eventually harm the body's systems [55]. Because there is lymph tissue throughout the body, the cancer cells may spread to other organs, or even into the bone marrow [55]. Recent experimental studies showed that profound mRNA expression changes of potential target genes involving cell cycle control, apoptosis and B-cell differentiation was concerned with the down-regulation of miR-16, miR-26a, miR-101, miR-29c and miR138 in the t(14;18)-negative follicular lymphoma subset [56]. Therefore, it is important to take lymphomas as a case to study. Through implementing SSCMDA for potential miRNA-disease association prediction, 6 out of top 10 potential lymphoma-associated miRNAs in the prediction result list have been verified by the recent studies. Furthermore, for the top 50 lymphoma-associated miRNAs predicted by SSCMDA, 39 of them have experimental literature evidences (See Table 4). For example, the hsa-mir-221 was ranked first in the prediction and it has been confirmed to have a significant difference in expression of lymphoid tissues between the lymphoma patient and healthy groups [57].

**Table 4:** Prediction of the top 50 predicted miRNAs associated with lymphoma based on known associations in HMDD database

miRNA	Evidence	miRNA	Evidence
hsa-mir-221	dbdemc;miR2Disease	hsa-mir-422a	dbdemc
hsa-mir-2355	unconfirmed	hsa-mir-17	dbdemc;miR2Disease
hsa-mir-1247	unconfirmed	hsa-mir-29c	dbdemc
hsa-mir-215	dbdemc	hsa-mir-222	dbdemc
hsa-mir-922	unconfirmed	hsa-mir-181c	dbdemc
hsa-mir-9	dbdemc	hsa-mir-26a	dbdemc
hsa-mir-202	unconfirmed	hsa-mir-183	dbdemc
hsa-mir-29b	dbdemc	hsa-mir-200b	dbdemc
hsa-mir-326	dbdemc	hsa-mir-92a	dbdemc
hsa-mir-1915	unconfirmed	hsa-mir-103a	unconfirmed
hsa-mir-10b	dbdemc	hsa-mir-204	dbdemc
hsa-let-7d	dbdemc	hsa-mir-139	dbdemc;miR2Disease
hsa-mir-1302	unconfirmed	hsa-mir-181a	dbdemc
hsa-mir-18a	dbdemc	hsa-mir-92	dbdemc;miR2Disease
hsa-mir-15b	dbdemc	hsa-mir-182	dbdemc
hsa-mir-99b	dbdemc	hsa-let-7a	dbdemc
hsa-let-7e	dbdemc;miR2Disease	hsa-mir-195	dbdemc
hsa-let-7f	dbdemc	hsa-mir-21	dbdemc;miR2Disease
hsa-mir-498	unconfirmed	hsa-mir-200a	dbdemc
hsa-let-7g	dbdemc	hsa-mir-33a	dbdemc
hsa-mir-518a	unconfirmed	hsa-mir-27a	dbdemc
hsa-let-7c	dbdemc	hsa-mir-181b	dbdemc
hsa-mir-10a	dbdemc;miR2Disease	hsa-mir-20a	dbdemc;miR2Disease
hsa-let-7b	dbdemc	hsa-mir-320e	unconfirmed
hsa-mir-483	unconfirmed	hsa-mir-31	dbdemc

The first column records top 1-25 related miRNAs. The second column records the top 26-50 related miRNAs.

For demonstrating all the prediction results, we showed the prediction scores of all the candidate miRNA-disease pairs in Supplementary Table 1. This table contains the potential miRNAs associated with all the human diseases investigated in HMDD database (See Supplementary Table 1).

### Case study for new disease in the simulation experiments

To validate the prediction effectiveness for new diseases, we also conducted another case study by hiding the information of miRNAs related with the investigated disease. For each investigated disease, we removed the known associations between this disease and all its related miRNAs. Through implementing the SSCMDA based on the changed input association matrix, we obtained the ranking of all the candidate miRNA-disease pairs by comparing their prediction scores. We could find that 9, 18 and 41 out of the top 10, 20 and 50 miRNAs of hepatocellular carcinoma had been confirmed by at least one of the three databases, namely HMDD, dbDEMC and miR2Disease (See Table 5). Specially, the has-miR-16, whose ranking is the first of the prediction result, has been studied by researchers and they found that the cyclooxygenase-2 whose expression has been detected in human hepatocellular carcinoma is a target of has-miR-16 [58].

**Table 5:** Prediction of the top 50 miRNAs associated with Hepatocellular Carcinoma in HMDD database

miRNA	Evidence	miRNA	Evidence
hsa-mir-16	dbdemc;miR2Disease;HMDD	hsa-mir-126	dbdemc;miR2Disease;HMDD
hsa-mir-182	miR2Disease;HMDD	hsa-mir-139	miR2Disease;HMDD
hsa-mir-143	dbdemc;miR2Disease	hsa-mir-1302	unconfirmed
hsa-mir-146a	dbdemc;miR2Disease;HMDD	hsa-mir-125a	dbdemc;miR2Disease;HMDD
hsa-mir-15a	dbdemc;miR2Disease;HMDD	hsa-mir-17	miR2Disease;HMDD
hsa-mir-382	unconfirmed	hsa-mir-26b	dbdemc;miR2Disease
hsa-mir-200c	HMDD	hsa-let-7i	dbdemc;HMDD
hsa-mir-107	dbdemc;miR2Disease;HMDD	hsa-mir-223	miR2Disease;HMDD
hsa-mir-345	HMDD	hsa-mir-922	unconfirmed
hsa-mir-103a	HMDD	hsa-let-7c	dbdemc;miR2Disease;HMDD
hsa-mir-200a	dbdemc;miR2Disease;HMDD	hsa-mir-1247	unconfirmed
hsa-mir-655	unconfirmed	hsa-mir-205	miR2Disease;HMDD
hsa-mir-141	miR2Disease;HMDD	hsa-mir-203	miR2Disease;HMDD
hsa-mir-152	miR2Disease;HMDD	hsa-mir-215	miR2Disease
hsa-let-7d	miR2Disease;HMDD	hsa-mir-29b	dbdemc;HMDD
hsa-mir-10b	HMDD	hsa-mir-148b	dbdemc;miR2Disease;HMDD
hsa-mir-92a	miR2Disease;HMDD	hsa-mir-1972	unconfirmed
hsa-let-7a	dbdemc;miR2Disease;HMDD	hsa-mir-191	dbdemc;HMDD
hsa-mir-181b	dbdemc;miR2Disease;HMDD	hsa-mir-106a	dbdemc;miR2Disease;HMDD
hsa-mir-20a	dbdemc;miR2Disease;HMDD	hsa-mir-100	dbdemc;HMDD
hsa-mir-132	miR2Disease	hsa-mir-299	unconfirmed
hsa-mir-194	dbdemc;miR2Disease	hsa-mir-195	dbdemc;miR2Disease;HMDD
hsa-mir-204	unconfirmed	hsa-mir-27b	dbdemc
hsa-mir-9	miR2Disease	hsa-mir-27a	miR2Disease;HMDD
hsa-mir-200b	miR2Disease;HMDD	hsa-mir-675	unconfirmed

The hepatocellular carcinoma was treated as new disease which does not have any related miRNAs. The first column records top 1-25 related miRNAs. The second column records the top 26-50 related miRNAs.

### Case study on previous version dataset

Finally, we tested our model based on the old version of the database HMDD to see whether the SSCMDA still performed well on it. Through the experiment, there were 7, 13 and 29 respectively out of top 10, 20 and 50 miRNAs related with the Glioblastoma confirmed by at least one of three databases mentioned above (See Table 6). Taking the hsa-mir-338 as an example, which ranked the first among all the miRNAs verified by dbDEMC or miR2Disease, recent research showed that the mir-338-3p inhibited malignant biological behaviors of Glioblastoma cells by targeting MACC1 gene [59].

**Table 6:** Prediction of the top 50 predicted miRNAs associated with Glioblastoma based on known associations in previous version HMDD database

miRNA	Evidence	miRNA	Evidence
hsa-mir-561	unconfirmed	hsa-mir-135b	dbdemc
hsa-mir-338	dbdemc	hsa-mir-194	dbdemc
hsa-mir-301a	unconfirmed	hsa-mir-423	unconfirmed
hsa-mir-342	dbdemc;HMDD	hsa-let-7b	unconfirmed
hsa-mir-25	dbdemc;miR2Disease;HMDD	hsa-mir-185	dbdemc
hsa-mir-499	unconfirmed	hsa-mir-525	unconfirmed
hsa-mir-370	dbdemc	hsa-let-7e	dbdemc
hsa-mir-134	HMDD	hsa-mir-532	unconfirmed
hsa-mir-23b	miR2Disease;HMDD	hsa-mir-218	dbdemc;HMDD
hsa-mir-421	dbdemc	hsa-mir-181c	dbdemc;miR2Disease;HMDD
hsa-mir-136	dbdemc	hsa-mir-514	unconfirmed
hsa-mir-34a	dbdemc;miR2Disease;HMDD	hsa-mir-518c	unconfirmed
hsa-mir-330	unconfirmed	hsa-mir-638	unconfirmed
hsa-mir-199b	dbdemc	hsa-mir-19a	dbdemc;HMDD
hsa-mir-583	unconfirmed	hsa-mir-335	unconfirmed
hsa-mir-30b	unconfirmed	hsa-mir-325	unconfirmed
hsa-mir-451	miR2Disease	hsa-mir-126	unconfirmed
hsa-mir-520f	unconfirmed	hsa-mir-383	dbdemc
hsa-mir-520c	dbdemc	hsa-mir-539	HMDD
hsa-mir-210	dbdemc;HMDD	hsa-mir-135a	unconfirmed
hsa-mir-29a	HMDD	hsa-mir-365	unconfirmed
hsa-mir-596	unconfirmed	hsa-mir-153	dbdemc;miR2Disease;HMDD
hsa-mir-300	dbdemc	hsa-mir-379	dbdemc
hsa-mir-206	dbdemc;HMDD	hsa-mir-382	dbdemc
hsa-mir-128a	miR2Disease	hsa-mir-130b	unconfirmed

The first column records top 1-25 related miRNAs. The second column records the top 26-50 related miRNAs.

## DISCUSSION

In this paper, we proposed the Spy and Super Cluster strategy for MiRNA-Disease Association prediction (SSCMDA) based on known miRNA-disease associations, integrated miRNA similarity and integrated disease similarity. The Regularized Least Square (RLS) was used as the baseline classifier. The spy strategy was implemented to identify the negative samples with high degree of confidence from the mixed sample set which contains potential associations and real negative associations. Because the shortage of positive samples would lead to lower accuracy of prediction, the super cluster strategy was conducted to increase as many positive samples as possible. To examine the accuracy of the SSCMDA, three types of cross validations including global LOOCV, local LOOCV and 5-fold cross validation had been implemented. Furthermore, we also implemented three types of case studies based on different miRNA-disease association databases. As a result, SSCMDA performed well both in the cross validations and the case studies.

The excellent performances of SSCMDA could be attributed to the following several important factors. Firstly, the increasing disease-miRNA association data have been discovered and confirmed with the development of the biological experiments. This advantage was beneficial to the SSCMDA because the SSCMDA was dependent on the known associations to improve the prediction accuracy. Secondly, because SSCMDA was constructed based on the integrated disease similarity and integrated miRNA similarity, it could make full use of various similarity information to recover the potential miRNA-disease associations. Thirdly, to improve the prediction accuracy, SSCMDA could adopt spy strategy to identify reliable negative samples from all the unknown miRNA-disease pairs, which contained mixed training samples including both potential associations and real negative samples. Meanwhile, the super cluster strategy could add as many positive samples as possible with the help of the similarity information. Last but not least, the baseline method of RLS performed well in previous computational biology research, which guaranteed the basic accuracy of our proposed model. In view of reasons mentioned above, SSCMDA could improve the prediction accuracy in comparison with the previous proposed methods.

However, there are still some limitations in this model. First of all, the proposed model relied heavily on the known association data, which will lead to an unstable prediction effect when the training samples were changed. Secondly, the RLS is so sensitive to the changes of parameters that it is hard to obtain the optimal combining parameters to combine all the proposed strategies to improve prediction accuracy. Therefore, the method of obtaining optimal combining parameters is expected to be theoretically solved in the future. Furthermore, the literature of Wang *et al*. has pointed out the more important fact that it is unwise to use a single disease-related miRNA to judge cancer risks for all the persons [60]. The recent study has also found that cancer signals are of not stable, which motivates us to construct various cancer hallmark networks to effectively evaluate cancer risks based on the miRNA profiles of each person. Furthermore, the re-sampling test should be developed in the future for estimating the robustness of the models [60, 61]. Additionally, there are still some important problems in the personalized medicine and treatment in the framework of miRNA-disease association prediction [60, 62]. According to the recent studies on the genome sequencing technology of cancer systems biology, we can anticipate that cancer subclones and other clone-based analysis would be helpful for predicting and treating cancer in the future [63, 64].

## MATERIALS AND METHODS

### Human miRNA-disease associations

We downloaded the human miRNA-disease associations data from the HMDD v2.0 database [65], which contained 5430 distinct experimentally confirmed human miRNA-disease associations between 383 diseases and 495 miRNAs. To conveniently deal with all these data in the following computations, we constructed an adjacency matrix *A* ε *R ^nd×nm^* to formalize the human miRNA-disease associations, where *nm* and *nd* were denoted as the number of miRNAs and diseases in HMDD v2.0 database, respectively. If miRNA *m_j_* had been experimentally verified to be associated with disease *d_i_*, then *A_ij_* equaled to 1, otherwise 0.

### MiRNA functional similarity

Based on the assumption that functionally similar miRNAs are more likely to be associated with diseases with similar characters, Wang *et al.* [66] proposed a method to calculate the miRNA functional similarity. We downloaded the miRNA functional similarity data from http://www.cuilab.cn/files/images/cuilab/misim.zip We denoted the matrix *MS* to represent the miRNA functional similarity. The element *MS_ij_* represented the value of similarity between the miRNA *m_i_* and *m_j_*.

### Disease semantic similarity model 1

We constructed a Directed Acyclic Graph (DAG) to describe the diseases according to the literature of Chen *et al.* [26] based on the MeSH descriptors downloaded from the National Library of Medicine (http://www.nlm.nih.gov/). According to the DAG, we denoted the contribution values of disease *d* in DAG(*D*) to the semantic value of disease *D* as follows:

$$\left\{ \begin{array}{l} D1_{D}\left(d\right)=1\;if\;d=D\\ D1_{D}\left(d\right)=\max\left\{\Delta *D1_{D}\left(d^{\prime }\right)|d^{\prime }\in children\;of\;d\right\}\;if\;d\neq D \end{array}\right.$$ (1)

where Δ was the semantic contribution decay factor. The self semantic value of disease *D* was defined as follows:

$$\text{D}V1\left(D\right)=\sum_{d\in T(D)}D1_{D}(d)$$ (2)

where *T*(*D*) represented all ancestor nodes of *D* and *D* itself. According to the observation that two diseases with larger shared part of their DAGs had larger similarity score, the semantic similarity score between disease *d_i_* and *d_j_* could be defined as follows:

$$SS1(d_{i},d_{j})=\frac{\sum_{t\in T(d_{i})\cap T(d_{j})}\left(D1_{d_{i}}(t)+D1_{d_{j}}(t)\right)}{DV1(d_{i})+DV1(d_{j})}$$ (3)

### Disease semantic similarity model 2

As what we had considered, it was not reasonable to assign the same contribution value to the diseases in the same layer of DAG(*D*). As far as we have observed, a more specific disease which appears in less DAGs should contribute a higher value to the semantic similarity of disease *D*. Therefore, according to the model which was proposed by Xuan *et al.* [33], we defined the contribution of disease *d* in DAG(*D*) to the semantic value of disease *D* as follows:

$$D2_{D}(d)=-\log[\text{the number of DAGs including t}/\text{the number of diseases}]$$ (4)

We defined the semantic similarity of disease *d_i_* and *d_j_* as the ratio of the shared ancestor nodes’ contributions to all the ancestor nodes’ contributions. Therefore, disease semantic similarity model 2 was calculated as follows:

$$SS2(d_{i},d_{j})=\frac{\sum_{t\in T(d_{i})\cap T(d_{j})}\left(D2_{d_{i}}(t)+D2_{d_{j}}(t)\right)}{DV2(d_{i})+DV2(d_{j})}$$ (5)

where

$$\text{D}V2\left(D\right)=\sum_{d\in T(D)}D2_{D}(d)$$ (6)

### Gaussian interaction profile kernel similarity

Based on the Gaussian kernel function which is one of the Radial Basis function whose value depends only on the distance from the origin, Gaussian interaction profile kernel similarity were constructed as another algorithm of disease semantic similarity and miRNA functional similarity [67]. As the *i^th^* row and *j^th^* column of adjacent matrix *A* contains the information whether the disease or the miRNA associated with each of the miRNAs or the diseases, so we denoted vector *IP*(*d_i_*) and *IP*(*r_j_*) to represent the *i^th^* row vector and *j^th^* column vector, respectively. Therefore, the similarity of diseases and miRNAs could be computed as follows:

$$GD(d_{i},d_{j})=\exp(-\beta_{d}{\left\|IP(d_{i})-IP(d_{j})\right\|}^{2})$$ (7)

$$GR(m_{i},m_{j})=\exp(-\beta_{m}{\left\|IP(m_{i})-IP(m_{j})\right\|}^{2})$$ (8)

where adjustment coefficient *β_d_* and *β_m_* for the kernel bandwidth were denoted as follows:

$$\beta_{d}=\beta {'}_{d}/\left(\frac{1}{nd}\sum_{i=1}^{nd}{\left\|IP(d_{i})\right\|}^{2}\right)$$ (9)

$$\beta_{m}=\beta {'}_{m}/\left(\frac{1}{nm}\sum_{i=1}^{nm}{\left\|IP(m_{i})\right\|}^{2}\right)$$ (10)

where *β′_d_* and *β′_m_* were the original bandwidths. In the end, matrix *GD* and *GR* represented the Gaussian interaction profile kernel similarity of diseases and miRNAs, respectively.

### Integrated similarity for miRNAs and diseases

Through combining the disease semantic similarity with disease Gaussian interaction profile kernel similarity, we obtained the integrated disease similarity. Specifically, if disease *d_i_* and *d_j_* have semantic similarity, then the final integrated similarity is the average of *SS1* and *SS2*, otherwise the integrated disease similarity equals to the value of Gaussian interaction profile kernel similarity. The formulations show as follows:

$$SD(d_{i},d_{j})=\left\{ \begin{array}{l} \frac{SS1(d_{i},d_{j})+SS2(d_{i},d_{j})}{2}\;d_{i}\text{ and }d_{j}\text{ has semantic similarity}\\ GD(d_{i},d_{j})\text{ otherwise} \end{array}\right.\;$$ (11)

Furthermore, by combining the miRNA functional similarity with miRNA Gaussian interaction profile kernel similarity, we obtained the integrated miRNA similarity as follows:

$$SR(m_{i},m_{j})=\left\{ \begin{array}{l} MS(m_{i},m_{j})\;m_{i}\text{ and }m_{j}\text{ has functional similarity}\\ GR(m_{i},m_{j})\text{ otherwise} \end{array}\right.\;$$ (12)

## SSCMDA

**Figure 2:**
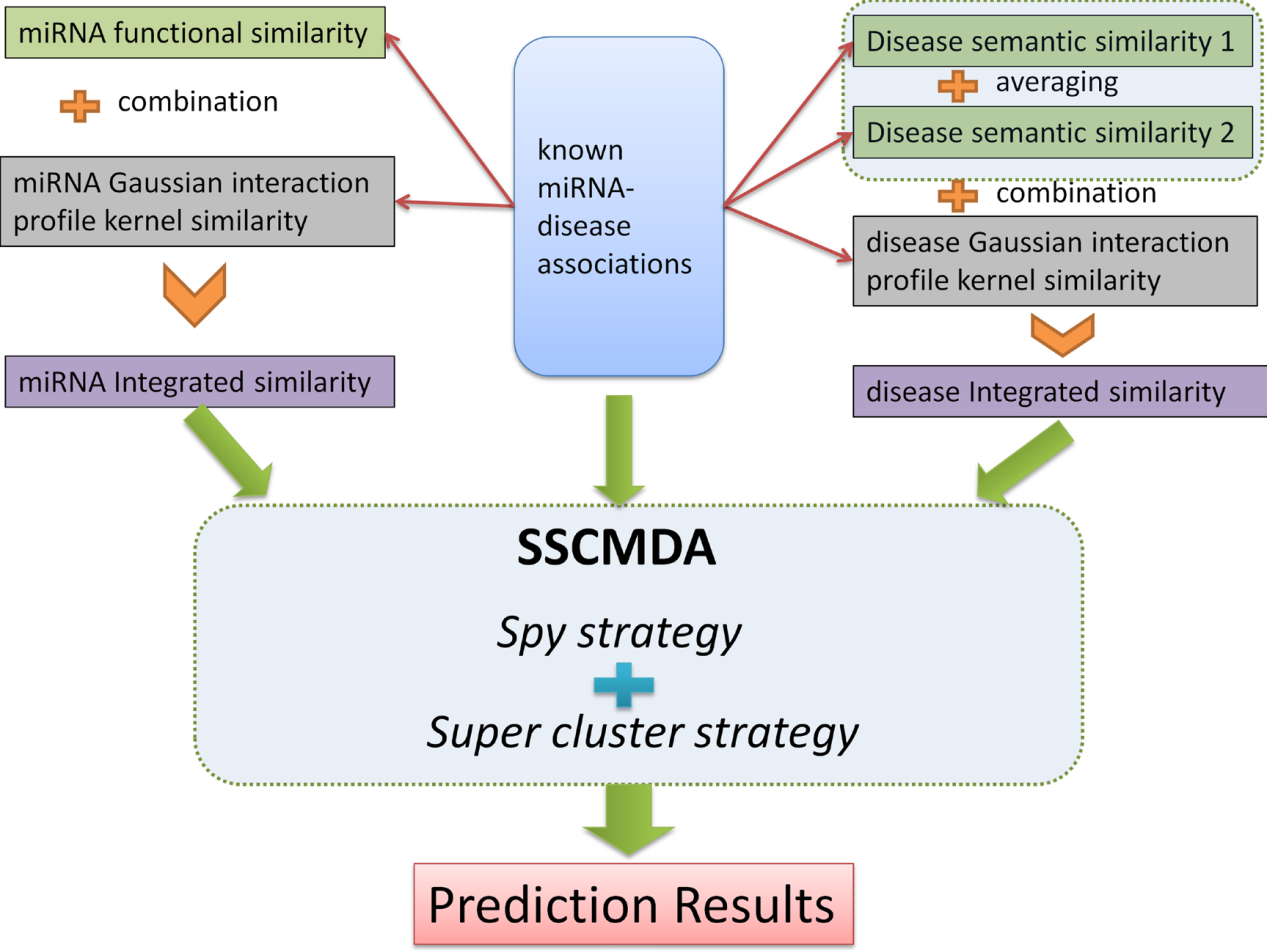
The whole process flowchart of the SSCMDA method The upper half part shows the input data including the known miRNA-disease associations, miRNA integrated similarity, and disease integrated similarity. The middle part shows the main algorithm including the Spy strategy and the Super cluster strategy. The final part is the prediction results.

The whole process of the proposed method was shown in Figure 2. Based on the materials prepared above, we adopted the RLS as our basic classifier, which was proposed to fit the known sample data and further predict the potential miRNA-disease associations [68]. According to the RLS and Lagrange multiplier method, we could construct our score function of association between disease *d_i_* and miRNA *m_j_* as follows:

$$S_{m_{j}}(d_{i})=SD_{i}{\left(SD+\lambda_{d}I\right)}^{-1}A^{T}{}_{j}$$ (13)

where S_*mj*_(*d_i_*) was the predicted association score of disease *d_i_* to miRNA *m_j_*, *SD_i_* was the *i^th^* row of the integrated disease similarity matrix, *λ_d_* was the regularization parameter rooting in the lagrangian multiplier of RLS and experimentally set as 2 which was a common set in the experiments, *I* represented the identity matrix and *A^T^_j_* represents the *nd×1* class label vector of training samples namely the association between all the diseases and the *j^th^* miRNA. On the other hand, the score function of miRNA to disease was as follows:

$$S_{d_{j}}(m_{i})=SR_{i}{\left(SR+\lambda_{r}I\right)}^{-1}A_{j}$$ (14)

where *S_dj_*(*m_i_*) was the predicted score of miRNA *m_i_* to disease *d_j_*, *SR_i_* was the *i^th^* row of the integrated miRNA similarity matrix, *λ_r_* was also a regularization parameter rooting in the lagrangian multiplier of RLS and experimentally set as 2 which was also a common set in the experiments. Differently, *A_j_* represented the *1×nm* vector which was the *j^th^* column of the adjacent matrix *A*. Afterwards, we combined the two score functions as follows:

$$FS(d_{i},m_{j})=\frac{S_{d_{i}}(m_{j})+S_{m_{j}}(d_{i})}{2}$$ (15)

where *FS*(*d_i_*, *m_j_*) was formed as the final basic score function based on the RLS.

### Spy strategy

**Figure 3:**
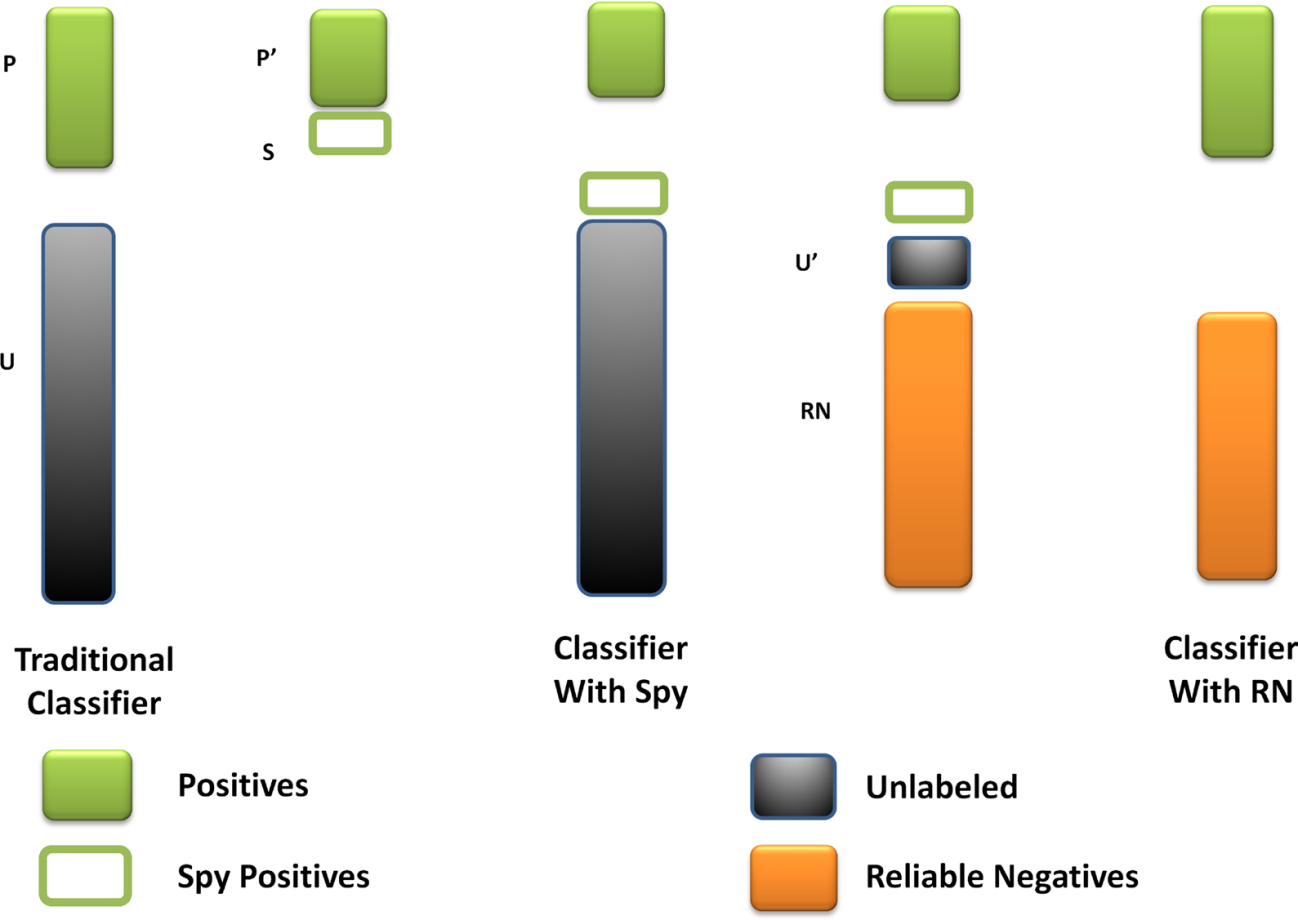
The flowchart of spy strategy Spy instances (**S**) are randomly selected from positives (**P**). The spy-based classifier is built by remaining positives (**P’**) and new unknown miRNA-disease pairs (**U’**) combined S with U. And the reliable negatives (**RN**) are identified by comparing with the minimum score of S.

As far as we had realized, the unknown miRNA-disease pairs contained both the potential associations and the real negative samples. The fuzzy situation would lead to an inaccurate prediction result because the boundary of negative training samples was not exact. To solve this problem, we utilized the spy strategy which was a semi-supervised strategy to identify the reliable negative samples with high degree of confidence from all the unknown miRNA-disease pairs. Specifically, we randomly selected 10% from the positive associations as spy samples, and set these spy samples to unlabeled ones, namely changed them from 1 to 0. Based on the newly formed training samples, we used the RLS model to obtain the prediction score. Then, we took the minimum score of the spy samples as the threshold. If the prediction score of a candidate association was below the threshold, this association would be identified as a reliable negative sample which would be set as -1 in the miRNA-disease association adjacent matrix. Repeating the selection of spy samples for 200 times, we took the intersection of the reliable negative sets as the final reliable negative set to ensure its reliability. Finally, we obtained a new adjacent matrix *AN* with all the reliable negative samples. Figure 3 illustrates the procession of spy strategy and the prediction score by adopting spy strategy could be calculated as follows:

$$FSpy(d_{i},m_{j})=\frac{Spy_{d_{i}}(m_{j})+Spy_{m_{j}}(d_{i})}{2}$$ (16)

where

$$Spy_{d_{j}}(m_{i})=SR_{i}{\left(SR+\lambda_{r}I\right)}^{-1}AN_{j}$$ (17)

$$Spy_{m_{j}}(d_{i})=SD_{i}{\left(SD+\lambda_{d}I\right)}^{-1}AN^{T}{}_{j}$$ (18)

### Super cluster strategy

**Figure 4:**
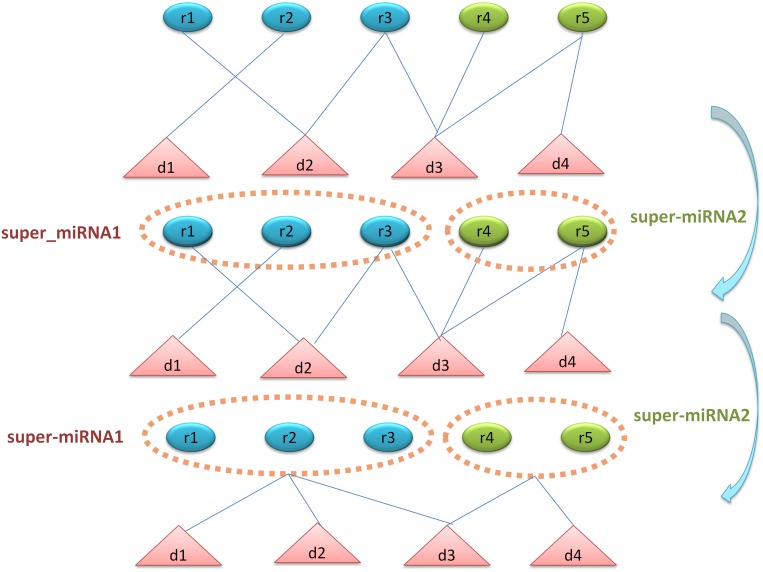
The flowchart of super-cluster strategy The first section shows the original known associations between diseases and miRNAs. The second section shows the process of constructing super-miRNAs based on miRNA functional similarity and agglomerative hierarchical clustering. The third section shows the process of constructing the new associations between diseases and super-miRNAs. If the disease has association with the miRNAs in the super-miRNA, then this disease is associated with the super-miRNA.

In consideration that most of the diseases might associate with only one or a few miRNAs and vice versa. This imbalance would lead to a biased prediction which was likely to determine potential associations as negative associations. The shortage of known miRNA-disease associations would aggravate this bias. Therefore, we proposed the super cluster strategy to ameliorate this problem (See Figure 4). The main thought of super cluster strategy is to cluster as many as possible similar diseases or miRNAs as an integrated super-disease or super-miRNA. Furthermore, all the diseases related to at least one miRNA in the super-miRNA would be seemed as having association with the super-miRNA. On the other hand, the association between miRNA and super-disease would be constructed in the same way. Obviously, the integrated similarity of miRNA and disease would be used to construct the distances between two miRNAs or two diseases. According to these distances, we implemented the agglomerative hierarchical clustering to obtain super cluster. The agglomerative hierarchical clustering adopted a bottom-up strategy which deemed each entity as a cluster at the beginning of the process, then one cluster would merge the other clusters based on the linkage criterion of Ward's minimum variance method [69]. After clustering, we cut the hierarchical clustering tree to obtain different super-clusters with a suitable threshold. However, there was a situation that diseases associated with miRNAs in super-miRNA are significantly different, which betrayed the assumption that similar diseases were likely to be associated with similar miRNAs. Therefore, we would remove the association between a disease associated with super-miRNA *sr* if there were not any of its *k* nearest neighbors simultaneously associated with *sr*. The probability score of disease *d_i_* associated with super-miRNA *sr_j_* could be calculated as follows:

$$S_{sr_{j}}(d_{i})=SD_{i}{\left(SD+\lambda_{d}I\right)}^{-1}Asr^{T}{}_{j}$$ (19)

where *Asr* was the new adjacent matrix formed according to the associations between diseases and super-miRNAs. Accordingly, the score of miRNA *m_i_* and super-disease *sd_j_* could be calculated as follows:

$$S_{sd_{j}}(m_{i})=SR_{i}{\left(SR+\lambda_{r}I\right)}^{-1}Asd_{j}$$ (20)

where *Asd* was the adjacent matrix of associations between miRNA and super-disease. The new associations are really not as accurate as the old associations. However, the main dilemma of us is not the accuracy of associations currently, but that the exact known association is rare, which prevented improving the accuracy of prediction. Thus, our aim is to excavate more useful information of the known associations based on which we can further separate the more likely miRNAs associated with disease from the mixed miRNAs. It is obvious that excessive sacrifices of known association accuracy will lead to predictive accuracy degradation. Therefore, it is a problem of clustering criterion to balance the relationship between the two situations. Fortunately, the experimental common clustering criterion used in other similar problems is still effective for our model. Furthermore, we also implemented the experiments to compare the accuracy of the model with and without the new associations, whose comparison results really reflect the effectiveness of this strategy to improve the prediction accuracy.

### Two layer prediction model

We integrated the spy strategy and super-cluster strategy to form a two-layer prediction model. For disease *d_i_* and miRNA *m_j_*, the prediction score obtained by adopting spy strategy and the prediction score obtained by adopting super-cluster strategy were combined as follows to compute the final miRNA-disease association prediction score:

$$TS(d_{i},m_{j})=\frac{FSpy(d_{i},m_{j};d_{i}\in sd_{p})S_{sd_{p}}(m_{j})+FSpy(d_{i},m_{j};m_{j}\in sr_{q})S_{sr_{q}}(d_{i})}{2}$$ (21)

where $FSpy(d_{i},m_{j};d_{i}\in sd_{p})$represented the prediction score of *d_i_* and *m_j_* by adopting spy strategy which satisfied that $d_{i}\in sd_{p}$and $FSpy(d_{i},m_{j};m_{j}\in sr_{q})$satisfied that $m_{j}\in sr_{q}$.

## SUPPLEMENTARY MATERIALS TABLE




