# Supplementary material for: SSCMDA: spy and super cluster strategy for MiRNA-disease association prediction
Source: Oncotarget. 2017 Dec 1;9(2):1826–42. doi: 10.18632/oncotarget.22812 (PMC5788602; doi:10.18632/oncotarget.22812)
Supplement: Supplementary file 1 [file oncotarget-09-1826-s001.pdf]

SSCMDA: spy and super cluster strategy for MiRNA-disease association prediction

SUPPLEMENTARY MATERIALS

Supplementary Table 1: We applied SSCMDA to prioritize all the candidate miRNA-disease pairs based on all the known miRNA-disease associations in HMDD database as training samples. See Supplementary_Table_1
